# Implementation of Self-Management Interventions in Cancer Survivors: Why Are We Not There Yet?

**DOI:** 10.1007/s13187-021-02021-2

**Published:** 2021-05-02

**Authors:** Ben Rimmer, Linda Sharp, on behalf of Ways Ahead study team

**Affiliations:** Population Health Sciences Institute, Newcastle University Centre for Cancer, Newcastle University, Ridley Building 1, Newcastle upon Tyne, NE1 7RU England UK

**Keywords:** Self-management, Cancer, Survivorship, Interventions

## Abstract

Despite the growing evidence base for supported self-management for the improvement of quality of life, there is a lack of widespread implementation of self-management interventions for cancer survivors. We propose five key areas that, if addressed, would optimise the development and evaluation of these interventions, namely: (1) improving intervention adaptability to different survivor populations; (2) establishing intervention acceptability (and feasibility); (3) ensuring systematic description of interventions, their content, and active ingredients; (4) conducting process evaluations; and (5) assessing cost-effectiveness. These areas are an essential prerequisite for translation of self-management interventions from research into routine cancer care.

## Introduction

Self-management is an individual’s awareness and active participation in their recovery, recuperation, and rehabilitation, to minimise treatment consequences and promote survival, health, and wellbeing. There is a growing evidence base suggesting that self-management interventions can improve various clinical, psychosocial, and economic outcomes — including quality of life, physical and psychological wellbeing, and healthcare utilisation — in people with cancer [1]. This is particularly true for supported self-management, as health professionals, family and friends, and fellow patients are instrumental to a patient’s ability to manage their condition [2].

However, although supported self-management is a key focus of cancer strategies and plans in some countries (e.g. UK and Australia), self-management interventions have not generally been adopted into routine practice. For example, when researchers tried to roll out Oncokompas, under a third of eligible sites adopted it [3]. This may be because it is unclear what implementation approaches and changes to care pathways are necessary to support and embed self-management interventions. However, beyond this, there are several areas, which, if addressed, could optimise the development, evaluation, and, consequently, implementation of self-management interventions.

## Adaptability

Self-management interventions have typically been developed for survivors of a specific cancer, often breast cancer [1], or involved mixed groups of survivors. Cancer is a complex chronic illness, meaning a ‘one size fits all’ approach is not appropriate. For example, (at least some of) the self-management needs of young adult survivors of childhood cancer, at risk of long-term cardiovascular disease; middle-aged people with a life-limiting glioma, experiencing seizures; and older men with urinary problems following prostate cancer treatment may be very different. Conversely, for many cancers, survivors may struggle to adjust to their new normal and may benefit from support to self-manage issues around healthcare, treatment, distress, and re-establishing social roles.

Behaviour change interventions targeted to a specific population, which are personalised and tailored to individual needs, are more likely to be effective [4]. Hence, it is important to understand and encompass the needs of specific cancers during intervention development. These needs are likely to change over time, so what is needed to self-manage soon after initial treatment and in the longer term may be different. Still, targeted, tailored, and personalised interventions for individuals with different cancers at different timepoints are at tension with the idea of widespread adoption and implementation across routine cancer care.

A self-management programme that is adaptable or flexible across people, cancers, and time may be more attractive to implement. But how might this be achieved? Self-management interventions are typically either adjustment- or problem-focused. Adjustment-focused interventions aid the general transition to survivorship by teaching self-management skills, such as problem solving, goal setting, action planning, healthcare communication, motivation, and confidence [5]. Problem-focused interventions enhance the survivor’s capability to manage specific cancer-related issues, such as pain and fatigue. An adaptable self-management programme could address both adjustment and problems, perhaps with ‘core’ elements around adjustment that apply across cancers and targeted problem-focused elements for cancer-specific needs. Development need not start de novo; rather, it may be possible to adapt existing effective interventions for content and context [6].

## Acceptability

In research studies, cancer survivors generally report a high perceived need for, and positive attitude towards, self-management [7]. However, without evaluation in ‘routine practice’, it is unclear whether interventions are widely acceptable. Trial participants are often highly selected, so what is acceptable to them may not be generalisable. Consequently, testing in diverse ‘real world’ survivor populations is required to ensure widespread acceptability.

Self-management is not, and should not be, the sole responsibility of the survivor. Health professionals have an influential role, as do family members, who often act as informal carers [2]. It is, therefore, crucial to consider how self-management is perceived by those closely involved in the survivor’s formal and informal care. Patients may expect health professionals to fulfil a comprehensive role, working together to identify and address support needs. However, health professionals may conceptualise self-management as the patient’s responsibility [8] and, even if they view their role positively, may lack training, skills, time, and other resources to actively and routinely *support* self-management. Hence, addressing — as part of intervention development and testing — acceptability to, and feasibility for, health professionals to facilitate self-management is critical. Family should also be integrated in intervention development. This will help ensure that consequent interventions are acceptable to those who use them, as well as those who deliver and/or support them. It will also facilitate the formation of partnerships between patients, providers, and family, which are needed to enable integration of interventions into cancer care [5].

## Description

Defining the core components and active ingredients of an intervention is critical to ensuring that mechanisms of action (see below) are understood, and interventions are replicable (and/or adaptable by others). Taylor et al. identified five core components of self-management, namely: (1) education about the condition; (2) psychological strategies to support adjustment; (3) treatment adherence support strategies; (4) practical support for daily and cancer-specific activities; and (5) social support [9]. This resulted in the PRISMS taxonomy, a 14-component framework to aid the design and description of self-management interventions [10].

However, few self-management interventions in cancer utilise the same combinations of core components [1], with a variety of components identified [11]. For many interventions, what the components comprise is not clearly described. Moreover, less than 50% of such interventions include a theoretical framework in their development [12], despite theories of social cognition being the basis for self-management. These limitations may help to account for the inconsistent evidence of effectiveness [8] and undoubtedly hinder understanding of what intervention components are (most) effective and, hence, translation into practice.

To enable progress in this area, those who develop interventions should describe the intervention and map the content and postulated active ingredients, using established frameworks, such as TIDiER [13], PRISMS [10], and the Behaviour Change Taxonomy (https://www.bct-taxonomy.com/about). Regarding implementation, this would help inform training and support requirements, so that the delivery of self-management interventions beyond the research context can be improved.

More generally, there is a need to synthesise the evidence from existing interventions to understand what they involve and whether specific features (i.e. characteristics, components, and behavioural change techniques) are associated with efficacy (in a trial context) and effectiveness (in the ‘real world’). Systematic intervention description would also support this.

## Process Evaluations

Process evaluations have a fundamental role in understanding how and for whom interventions do or do not work [14]. However, few, if any, process evaluations of self-management interventions in cancer have been conducted. Thus, evidence is lacking on which intervention components improve outcomes (and by what mechanisms), and the contextual factors that influence this. Likewise, for ineffective interventions, it is unclear whether this is because fidelity was poor, or mechanisms of action were incorrectly specified.

Process evaluations can serve different functions at each stage of intervention development and evaluation. Evaluation at the pilot stage could inform acceptability of implementation, while evaluation at the effectiveness stage might inform the function of different mechanisms [14]. Importantly, the evaluation of each development stage must consider all stakeholders, including survivors, family, health professionals, and commissioners/providers.

## Cost-Effectiveness

Evidence from various chronic diseases suggests that self-management can reduce healthcare resource utilisation [15]. Conversely, implementing self-management support may require the healthcare organisation to provide training, time, and material resources [9].

However, there is limited evidence to indicate whether implementing a self-management intervention into routine cancer practice is cost-effective. Most trials have failed to report any health economic evaluation. In the instance of two interventions, reported cost-effectiveness was not entirely conclusive [11]. A pertinent issue for economic evaluations to date is the short time horizon, as actual costs and benefits are likely to accrue over a long time.

The real-world implementation of a self-management intervention requires a whole-system approach [9]. Evidence on cost-effectiveness and budgetary impact is expectedly critical for policy makers and service providers. Improvements to outcome measures are insufficient if they are marginal compared to operational costs of the intervention.

## Conclusion

Future research on self-management interventions in cancer survivors should consider and incorporate adaptability across people, cancers, and time; acceptability to end users, including survivors, family, and health professionals; systematic descriptions of the intervention, its content, and active ingredients; process evaluations to understand what does and does not work; and evaluation of cost-effectiveness. These are essential for rigorous, systematic, and transparent intervention development, specification, testing, and reporting and are a prerequisite for translation from research into routine cancer care.

## Data Availability

Data sharing is not applicable to this article as no new data were created or analysed.
